# Elevated plasma homocysteine upon ischemic stroke is associated with increased long-term mortality in women

**DOI:** 10.1371/journal.pone.0183571

**Published:** 2017-08-28

**Authors:** Ioanna Markaki, Stefanos Klironomos, Konstantinos Kostulas, Christina Sjostrand

**Affiliations:** Department of Clinical Neuroscience, Karolinska Institutet, Stockholm, Sweden; Medizinische Universitat Innsbruck, AUSTRIA

## Abstract

**Background:**

Ischemic stroke is a leading cause of death worldwide, despite preventive and therapeutic advances during the last twenty years. Blood-borne biomarkers have been studied in association to short- and long-term outcome, in order to investigate possible modifiable predictors of disability and death. Increased homocysteine has been associated with increased vascular risk and unfavorable outcome, but homocysteine lowering treatment has not consistently been successful in risk reduction. The aim of this study was to investigate homocysteine levels upon acute ischemic stroke in association to long-term mortality.

**Methods:**

Of 622 patients included in our hospital-based registry, 331 survived the first month after admission, and had a diagnosis of ischemic stroke and available homocysteine values. All-cause and vascular mortality were investigated based on the national patient- and cause of death-registries. Survival analysis and Cox proportional hazard models were used to investigate time to death and predictors of outcome.

**Results:**

Of 331 patients, 148 (45%) had low homocysteine (<13 micromol/L) and 183 (55%) had high homocysteine (> = 13 micromol/L). During 10 years of follow-up (median 5.5 years), 47 patients (32%) with low homocysteine and 94 (51%) with high homocysteine died (p<0.0001). Estimated median survival was not reached for the low homocysteine group, and was 80 months in the high homocysteine group (p with log-rank test 0.002). High homocysteine was not independently associated with increased risk for death after adjustment for age, sex, comorbidities, and eGFR (HR 1.29, 95% CI 0.86–1.93; p = 0.2). Subgroup analysis by sex showed that high homocysteine was an independent predictor of mortality in women after adjustment for age and vascular comorbidities (HR 1.85; 95% CI 1.03–3.31; p = 0.04), but not in men (HR 0.87; 95% CI 0.52–1.43; p = 0.6).

**Conclusion:**

Increased plasma homocysteine (> = 13 micromol/L) upon acute ischemic stroke was not independently associated with mortality in our study. In the subgroup of women, high homocysteine was associated with increased five-year risk of death. Our study’s retrospective design and the exploratory nature of subgroup analysis, prevent robust conclusions based on that observation. Future studies on homocysteine levels before as well as upon stroke will shed further light on a possible causal association.

## Introduction

Stroke is the second most common cause of death worldwide [1], and despite a declining trend of age-standardized mortality rates during the last twenty years, the absolute number of stroke-related deaths and the global burden of stroke are great and increasing [2]. Age, stroke severity, diabetes, history of dementia and malignancy, as well as renal failure, peripheral atherosclerosis and chronic heart failure, are all predictors of long-term mortality that can only partly if at all be reversed [3]. Blood-borne biomarkers have been suggested as useful, potentially modifiable predictors of ageing processes, appropriate for interventional studies designed to improve health span [4]. In a large systematic review, C-reactive protein, white blood cell count, and N-Terminal pro Brain Natriuretic Peptide were strongly associated with all-cause and cardiovascular mortality, which was also shown for cholesterol fractions, fibrinogen and total homocysteine (tHcy), however with more limited evidence [5]. Numerous studies have demonstrated that elevated tHcy is an independent predictor of coronary heart disease and stroke risk (reviewed in [6]). A recent, well-designed, prospective study has also shown that elevated tHcy in the acute phase of ischemic stroke was associated with increased 2-year mortality, especially in patients with large vessel atherosclerosis [7]. tHcy -lowering treatment has not been consistently shown to reduce the risk for recurrent ischemic events and death [8], however selected subgroups of patients seem to benefit from B-vitamin substitution against stroke risk [9, 10].

In our study, we aimed to further investigate the association between tHcy status upon acute ischemic stroke and long-term mortality over ten years of follow-up.

## Materials and methods

The study was approved by the Regional Ethical Review Board in Stockholm. The Karolinska University Hospital in Huddinge is a hospital of reference with approximately 800 beds, serving an ethnically diverse population of around 250,000 persons. We aimed to design a hospital-based register on cerebrovascular diseases based on a standardized protocol including demographic characteristics, vascular risk factors, comorbidities, clinical investigation results, biochemical and neuroimaging data, complications, and outcome. Since 2006, all medical records including previous and current medication and laboratory results are digitalized. Our study was designed as a retrospective, hospital-based, follow-up cohort including consecutive patients admitted in 2006 and 2010, and followed until the 30^th^ of May, 2016.

Of 622 patients enrolled in the register, 193 were excluded due to diagnoses others than ischemic stroke (intracerebral hemorrhage, n = 69; transient ischemic attack, n = 109; other cerebrovascular disease, n = 19). Patients that did not survive the first month after ischemic stroke (n = 31) were excluded from the present analysis. Data on mortality and cause of death during the follow-up period were available for all patients through the national patient- and cause of death- registries. The primary outcome measure was all-cause mortality, and the secondary outcome measure was the composite end-point vascular mortality (ICD-10 codes I21, I24, I25, I26, I46, I49, I50, I51, I61, I62, I63, I64, I66, I67, I69, I70, I71, I97) and recurrent ischemic stroke or myocardial infarction (I63, I21). Clinical and biochemical data were obtained by reviewing medical records of all patients. Comorbidities were considered present based on the diagnoses registered in patient-records, if patients had ongoing treatment for the specific disease, or if newly diagnosed during hospital stay for the index stroke. All blood samples were drawn at admission, except for HbA1c, tHcy and cholesterol that were taken 8–12 hours after overnight fasting. Estimated glomerular filtration rate (eGFR) was calculated by the MDRD equation [186 x (Creatinine / 88.4)^-1.154^ x (Age)^-0.203^ x (0.742 if female) x (1.210 if black)]. tHcy was measured in the routine laboratory with enzymatic reaction, photometric assay, with reference range 5–15 μmol/L.

### Statistical analysis

Baseline characteristics were compared with the chi-square test for categorical variables, t test for normally distributed continuous variables (eGFR, erythrocyte count, hemoglobin, and total cholesterol; expressed as means ± standard deviation), and Wilcoxon-Mann-Whitney test for the remaining, non-normally distributed, continuous variables (expressed as medians with the interquartile range into parenthesis). Kaplan-Meier survival curves were produced for each tHcy quartile, and formed two clusters illustrating worse survival in patients that belonged in the higher tHcy quartiles compared to those in the first and second quartiles. For that reason, further analysis was performed after dichotomization of tHcy in the 50^th^ percentile (13 μmol/L), to evaluate this difference. Adjusted hazard ratios for mortality at 10 years were calculated with Cox proportional hazard model. Atherosclerosis was introduced as an independent variable, and included positive history of angina, myocardial infarction, claudication, revascularization or carotid stenosis. Eight patients were excluded from the final model due to missing values in comorbidities (atrial fibrillation, n = 1; heart failure, n = 1; diabetes mellitus, n = 5; malignant disease and dementia, n = 2). The proportional hazard assumption was validated using Schoenfeld residuals and the global test, and the goodness of fit of the final model was evaluated with the Cox-Snell residuals. Interaction between sex and tHcy status was tested in the multivariate model by setting male sex with low tHcy as the reference group, and the results included the hazard ratios for the subgroups: male sex with high tHcy, female sex with low tHcy and female sex with high tHcy. No correction for multiple comparisons has been done for the subgroup analysis of men and women, and thus it should be considered exploratory. Stata 12 software was used for the statistical analysis.

## Results

Of 398 patients that survived the first month post ischemic stroke, 331 had available tHcy values. Of those, 148 (45%) had low tHcy (<13 μmol/L) and 183 (55%) had high tHcy (> = 13 μmol/L). The range of tHcy values among patients was 4.7 to 64 μmol/L. Patients with high tHcy were older (median age: 76 vs. 69 years, p = 0.0001), had more often a history of myocardial infarction (21% vs. 12%, p = 0.03), and men were slightly overrepresented (63% vs. 55%, p = 0.1). Also, as presented in Table 1, patients with high tHcy, had higher creatinine and lower eGFR (median: 88 μmol/L vs. 69 μmol/L and mean 69 vs. 93 ml/min/1.73m^2^ respectively; p<0.0001), higher potassium (median: 4.1 mmol/L vs. 4 mmol/L; p = 0.0002), and lower glucose (median: 6.3 mmol/L vs. 7 mmol/L; p = 0.001).

**Table 1:** Baseline characteristics of study participants according to plasma homocysteine levels.

	Low tHcy (<13 μmol/L n = 148)	High tHcy (> = 13 μmol/L n = 183)	p
Age, years	69 (18)	76 (17)	<0.0001
Male sex	81 (55)	115 (63)	0.1
Comorbidities			
AF	41 (28)	49 (27)	0.9
Hypertension	93 (63)	130 (71)	0.1
Heart failure	16 (11)	32 (18)	0.08
Carotid stenosis	9 (6)	13 (7)	0.7
Angina	27 (18)	37 (20)	0.6
MI	18 (12)	39 (21)	0.03
CABG	9 (6)	11 (6)	0.9
PCI	3 (2)	9 (5)	0.2
Intermittent Claudication	3 (2)	7 (4)	0.5
DM	38 (26)	41 (23)	0.5
Hyperlipidemia	50 (34)	48 (27)	0.1
Hypothyroidism	18 (12)	9 (5)	0.02
COPD	9 (6)	13 (7)	0.7
Asthma	8 (5)	11 (6)	0.8
Cancer	18 (12)	24 (13)	0.8
DVT/LE	6 (4)	8 (4)	0.9
Migraine	8 (5)	4 (2)	0.1
Depression	7 (5)	8 (4)	0.9
Dementia	3 (2)	8 (4)	0.4
Sleep apnea	5 (3)	1 (1)	0.09
Smoking (ever)	56 (42)	90 (54)	0.5
Biochemical parameters			
Glucose, mmol/L	7 (3.9)	6.3 (1.9)	0.002
HbA1c, mmol/mol	42 (13)	41 (8)	0.9
Creatinine, μmol/L	69 (20)	88 (40)	<0.0001
eGFR, ml/min/1.73m^2^	93±25	69±28	<0.0001
White blood cells, x10^9^/L	8 (3.3)	8 (3)	0.7
Erythrocytes, x10^12^/L	4.6±0.5	4.45±0.6	0.03
Hemoglobin, g/L	140±15	138±17	0.3
Thrombocytes, x10^9^/L	230 (92)	225 (84)	0.1
Sodium, mmol/L	141 (4)	141 (4)	0.9
Potassium, mmol/L	4 (0.4)	4.1 (0.5)	0.0002
APTT, seconds	32.5 (5)	34 (6)	0.008
INR	1 (0.1)	1 (0.1)	0.7
Cholesterol, mmol/L	4.6±1.1	4.7±1.1	0.8
HDL cholesterol, mmol/L	1.3 (0.5)	1.2 (0.5)	0.3
LDL cholesterol, mmol/L	2.8 (1.4)	2.6 (1.4)	0.5
Triglycerides, mmol/L	1.15 (0.9)	1.3 (0.8)	0.05

AF, atrial fibrillation; MI, myocardial infarction; CABG, coronary artery bypass grafting; PCI, percutaneous coronary intervention; DM, diabetes mellitus; COPD, chronic obstructive pulmonary disease; DVT, deep venous thrombosis; LE, lung embolism; HbA1c, glycated hemoglobin; eGFR, estimated glomerular filtration rate; APTT, activated partial thromboplastin time; INR, international normalized ratio; HDL, high density lipoprotein; LDL, low density lipoprotein.

During 10 years of follow-up (median 5.5 years), 47 patients (32%) with low tHcy and 94 (51%) with high tHcy died (p<0.0001). Vascular diseases accounted for 50% vs. 42.5% of deaths in patients with high and low tHcy respectively. Infections and malignancies caused 17% and 11% of deaths respectively in the low tHcy group, and 6% and 15% of deaths in the high tHcy group. Dementia was the identified cause of death for 5 patients with high tHcy (5%), but for no patient with low tHcy. Other or unspecified causes of death accounted for 25.5% vs. 22% of deceased patients in the low and high tHcy group respectively.

**Fig 1:**
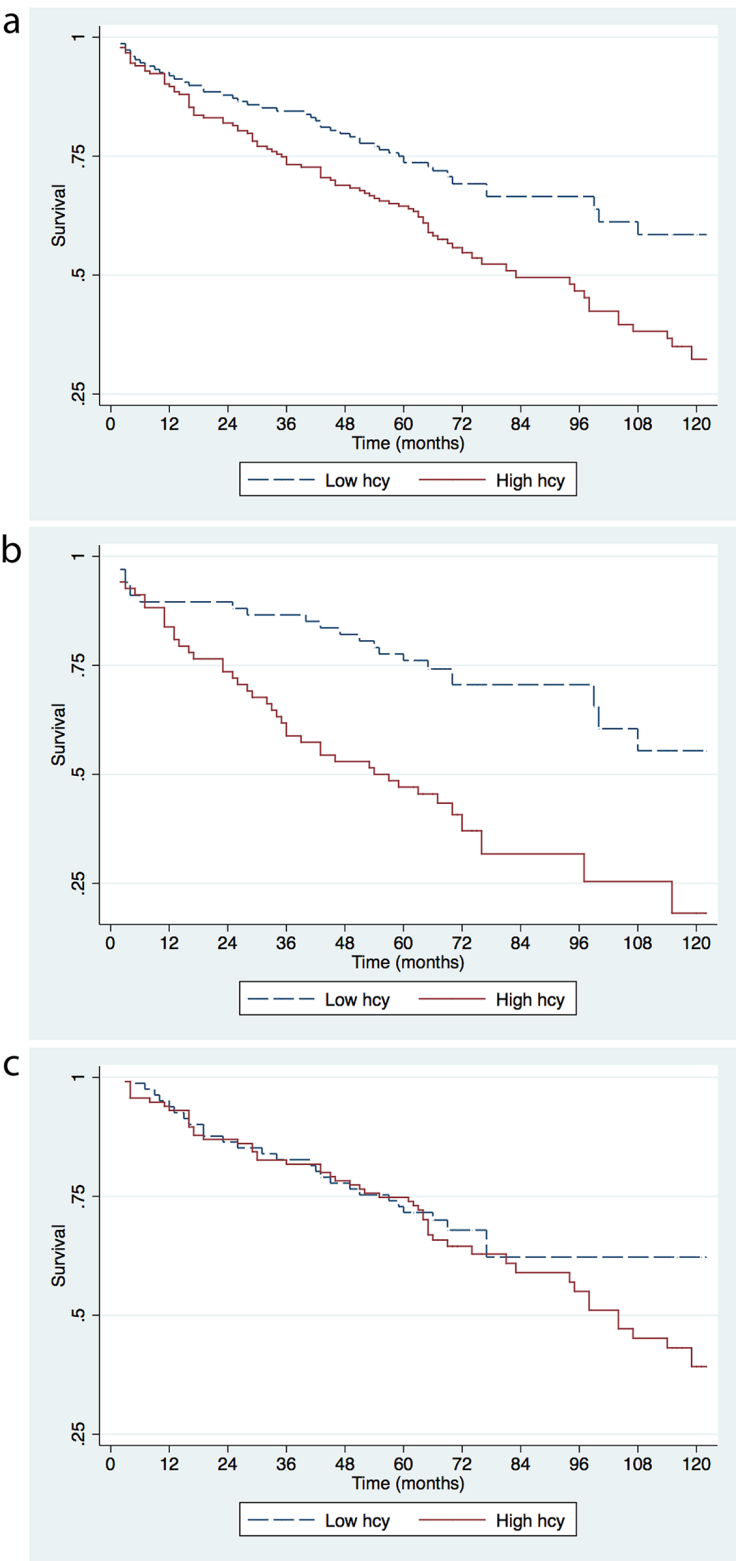
Kaplan-Meier survival curves for 10-year observation of 1-month survivors by tHcy status. The analyses were performed for all patients (a), for women (b), and men (c).

Survival curves of the two groups are presented in Fig 1a. Estimated median survival was not reached for the low tHcy group, and was 80 months in the high tHcy group (p with log-rank test 0.002). Further investigation of subgroups defined by sex showed that the difference was most pronounced among women (Fig 1b; p = 0.0001), whereas in men, there was no difference in survival between high and low tHcy groups (Fig 1c; p = 0.3).

Cox proportional hazard analysis showed that high tHcy was associated with increased risk for death (HR 1.7; 95% CI 1.2–2.4; p = 0.002) in the univariate analysis. However, the association was not statistically significant after adjustment for age, sex, and presence of atherosclerosis, atrial fibrillation, diabetes mellitus, malignancy, dementia, and eGFR (HR 1.29, 95% CI 0.86–1.93; p = 0.2). Subgroup analysis by sex showed that high tHcy was an independent predictor of mortality in women after adjustment for age and vascular comorbidities (HR 1.85; 95% CI 1.03–3.31; p = 0.04), but not in men (HR 0.87; 95% CI 0.52–1.43; p = 0.6). Interaction between sex and tHcy status was investigated in the multivariate model, and was not significant (HR for men with high tHcy 1.07; 95% CI 0.64–1.79; p = 0.8; HR for women with low tHcy 0.99; 95% CI 0.53–1.84; p = 0.9; HR for women with high tHcy 1.6; 95% CI 0.91–2.84; p = 0.1).

Competing risks analysis did now show independent association between the composite outcome vascular mortality/recurrent ischemic stroke or myocardial infarction and tHcy status, but the number of events was low for thorough subgroup investigation.

## Discussion

Our results demonstrate that increased plasma tHcy upon acute ischemic stroke is associated with increased long-term mortality, in a hospital-based cohort. Multivariate analysis showed that the association was not statistically significant after adjustment for age, sex, eGFR and comorbidities. However, in subgroup analysis, increased tHcy (> = 13 μmol/L) was independently associated with 85% increased risk of death five years after the event in women. No difference in survival was found in men with high vs. low tHcy levels. Examination of the causes of death showed higher mortality due to dementia, and cardiovascular and malignant diseases in patients with high tHcy compared to those with low tHcy levels. Our results may indicate a detrimental effect of increased tHcy in women with ischemic stroke, or an increase of tHcy in women with severe stroke but not in men.

Previous studies on tHcy and stroke outcome have mainly focused on short-term mortality. In a recent, multi-center, prospective Chinese cohort, women with higher baseline tHcy levels had higher risk of death and major disability three months after ischemic stroke, whereas no difference was observed in men [11]. Another study, in elderly patients, showed that moderate hyperhomocysteinemia (> = 30 μmol/L) was associated with poor functional status at discharge from the stroke unit, but not with early death [12]. Long-term outcome was examined in a large, prospective study where increased tHcy was associated with higher 4-year mortality in those with large vessel atherosclerosis, but not in patients with small vessel disease [13]. Patients with cardioembolic stroke, where women are overrepresented, were not reported in the latter study. Also, in a prospective longitudinal study in healthy, older persons (mean age: 75 years) in the Netherlands, elevated plasma tHcy was associated with increased risk for nursing home admission and mortality in women, but not in men [14].

Our study is a hospital-based cohort, with the shortcomings of a retrospective investigation, including missing data and information bias. Also, severity of stroke upon admission was not available to be taken into account for its impact on mortality. However, patients that did not survive the first month after ischemic stroke were excluded from the current analysis. Also, data on B-vitamin supplementation and other medication that can affect tHcy levels were not available. However, there is no evidence that statins [15], and other medication that is commonly prescribed among elderly [16] affect tHcy levels to a clinically relevant extend. Subgroup analysis separately for men and women is exploratory, and we advise caution with the interpretation of our results. Finally, renal function is an important factor influencing tHcy levels [17, 18], and it was included in our multivariate model. We also tested to exclude patients with renal insufficiency (eGFR < 15 ml/min/1.73m^2^, n = 3) and their exclusion did not change the results.

Overall, our study does not show an independent association of tHcy with all-cause mortality. In the subgroup of women, increased tHcy was associated with 85% increased risk of death. Potential causality cannot be established with the design of our study, and prospective interventional studies would be of interest to investigate whether treatment of hyperhomcysteinemia influences long-term outcome. Also, future studies that will evaluate tHcy levels before as well as upon ischemic stroke will answer the question whether tHcy is a marker of severe disease or a predictor of worse outcome in those with steadily increased levels.
